# Mandatory role of HMGA1 in human airway epithelial normal differentiation and post-injury regeneration

**DOI:** 10.18632/oncotarget.24511

**Published:** 2018-02-16

**Authors:** Haijun Zhang, Jing Yang, Matthew S. Walters, Michelle R. Staudt, Yael Strulovici-Barel, Jacqueline Salit, Jason G. Mezey, Philip L. Leopold, Ronald G. Crystal

**Affiliations:** ^1^ Department of Genetic Medicine, Weill Cornell Medical College, New York, NY, USA; ^2^ Department of Biological Statistics and Computational Biology, Cornell University, Ithaca, NY, USA

**Keywords:** HMGA1, regeneration, differentiation, wound repair, COPD

## Abstract

Due to high levels of expression in aggressive tumors, high mobility group AT-hook 1 (HMGA1) has recently attracted attention as a potential anti-tumor target. However, HMGA1 is also expressed in normal somatic progenitor cells, raising the question: how might systemic anti-HMGA1 therapies affect the structure and function of normal tissue differentiation? In the present study, RNA sequencing data demonstrated HMGA1 is highly expressed in human airway basal stem/progenitor cells (BC), but decreases with BC differentiation in air-liquid interface cultures (ALI). BC collected from nonsmokers, healthy smokers, and smokers with chronic obstructive pulmonary disease (COPD) displayed a range of HMGA1 expression levels. Low initial expression levels of HMGA1 in BC were associated with decreased ability to maintain a differentiated ALI epithelium. HMGA1 down-regulation in BC diminished BC proliferation, suppressed gene expression related to normal proliferation and differentiation, decreased airway epithelial resistance, suppressed junctional and cell polarity gene expression, and delayed wound closure of airway epithelium following injury. Furthermore, silencing of HMGA1 in airway BC in ALI increased the expression of genes associated with airway remodeling in COPD including squamous, epithelial-mesenchymal transition (EMT), and inflammatory genes. Together, the data suggests HMGA1 plays a central role in normal airway differentiation, and thus caution should be used to monitor airway epithelial structure and function in the context of systemic HMGA1-targeted therapies.

## INTRODUCTION

High mobility group AT-hook1 (HMGA1) codes for a 10 kDa protein that functions as a non-histone chromatin protein [1–3]. HMGA1 has three AT-hooks, each with an Arg-Gly-Arg-Pro core motif; using the AT-hooks, HMGA1 preferentially binds to the minor grooves of AT-rich regions in double stranded DNA [3–6]. HMGA1 plays a role in multiple cellular processes including replication and transcription [3, 7–10].

HMGA1 has recently become an anti-tumor target due to the correlation of high HMGA1 expression with tumor aggression and mortality [11–19]. A variety of anti-HMGA1 strategies, including small molecules, anti-sense mRNA expression, miRNA expression, and siRNA delivery, have demonstrated promising anti-tumor effects using transformed cells in *in vitro* systems and xenograft models [20–42]. However, systemic use of anti-HMGA1 therapeutics may affect HMGA1 expression in non-malignant cells, and the consequences of reduced HMGA1 expression in human somatic progenitor cells have not been studied.

Human airway basal cells (BC) are the stem/progenitor cells in the airway epithelium [43, 44]. Dysregulation of airway BC function has been linked to the pathogenesis of many lung diseases including chronic obstructive pulmonary disease (COPD) [45, 46]. Understanding the mechanisms that regulate BC differentiation and function are important to determine the pathogenesis of these diseases. Based on the knowledge that HMGA1 is highly expressed in airway BC, any therapy directed toward reducing HMGA1 expression or function should be evaluated with respect to normal airway physiology. In the present study, we used an *in vitro* culture system that models the biology of the airway [47–49] and an siRNA-based strategy to reduce HMGA1 expression levels in a manner consistent with several proposed therapeutic strategies [21, 22, 25, 31, 36, 50]. We evaluated the effect of reduced HMGA1 expression in airway BC on normal cell differentiation, differentiated airway epithelium function, and wound healing. The data demonstrates that the expression of HMGA1 is essential for airway BC proliferation, differentiation, airway barrier integrity, and wound repair. Interestingly, suppression of HMGA1 expression results in up-regulation of genes associated with abnormal BC differentiation to squamous, inflammatory and epithelial-mesenchymal transition (EMT) phenotypes, commonly observed in association with cigarette smoking and COPD [45, 46, 51].

## RESULTS

### HMGA1 expression in normal human airway basal cells

**Figure 1:**
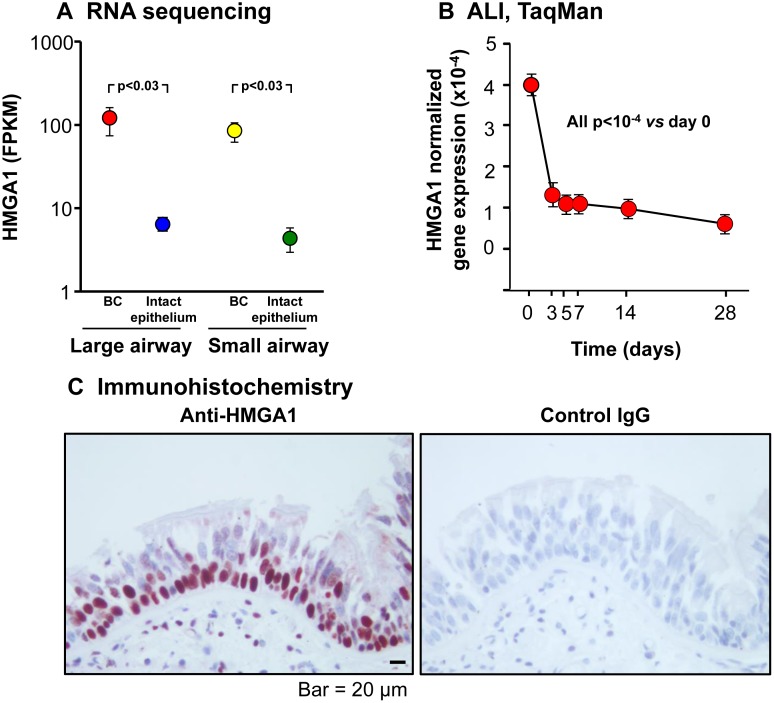
HMGA1 expression in intact large (LAE) and small airway epithelium (SAE), basal cells (BC) derived from LAE and SAE, and BC differentiated on air-liquid interface (ALI) culture (**A**) HMGA1 expression in LAE and SAE BC and intact LAE and SAE. Human LAE (3rd-4th order bronchi) of healthy nonsmokers and SAE (10th-12th order) of healthy nonsmokers was obtained by fiberoptic bronchoscopy by brushing. Intact epithelium was directly processed for RNA sequencing (LAE *n* = 10, SAE *n* = 22). BC were purified (LAE BC *n* = 20, SAE BC *n* = 3) as described in Methods (BC-1). (**B**) HMGA1 expression over time in ALI culture. Human LAE BC from Lonza were cultured in ALI (see Methods, BC-1 and ALI-1), and RNA harvested at ALI day 0-28. HMGA1 expression (TaqMan) was normalized to 18S rRNA. *n* = 4 each time point. (**C**) HMGA1 expression in a normal human LAE assessed by immunohistochemistry (left – HMGA1, right – control IgG).

We first characterized purified BC from brushed samples by immunohistochemistry using cell type-specific markers: KRT5 for BC, MUC5AC for secretory cells and TUBB4 for ciliated cells. BC phenotype was confirmed by positive staining for BC-specific marker KRT5 and negative staining for markers of different types of differentiated cells including MUC5AC and TUBB4 (Supplementary Figure 1). Analysis of the transcriptome of purified normal human large and small airway BC demonstrated high levels of HMGA1 expression, much greater than in the intact, differentiated airway epithelium (Figure 1A). Consistent with this data, HMGA1 expression during airway epithelial differentiation in ALI culture markedly decreased (all *p <* 10^−4^ *vs* day 0; Figure 1B), and immunohistochemistry assessment of HMGA1 expression in the human airway epithelium demonstrated high expression of HMGA1 in the BC layers, with less expression in intermediate cells, but not in the differentiated airway epithelial cells (Figure 1C).

### Low expression of HMGA1 in BC is associated with decreased regeneration capacity

**Figure 2:**
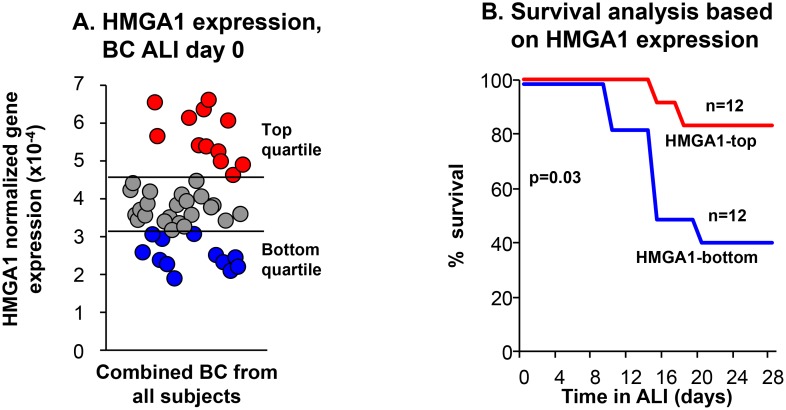
Relationship of BC expression of HMGA1 to the ability of SAE BC to differentiate Primary BC purified from human SAE sampled by bronchoscopic brushing of 47 individuals (17 nonsmokers, 14 healthy smokers and 16 COPD smokers). (**A**) HMGA1 expression evaluated by TaqMan at day 0 before differentiation on ALI culture (see Methods, BC-1 and ALI-1). Based on the data in panel A, samples were categorized as “top HMGA1” expressors (HMGA1 expression highest quartile, *n* = 12), and “bottom HMGA1” expressors (HMGA1 expression lowest quartile, *n* = 12). The data was normalized to 18S rRNA. (**B**) Kaplan–Meier survival analysis on ALI cultures for HMGA1-top and –bottom quartile expressors of all nonsmokers, healthy smokers and COPD smokers in SAE BC samples. *p* = 0.03, log-rank test.

We previously observed that airway BC from healthy smokers and COPD smokers have “basal cell fatigue,” a diminished capacity to regenerate airway epithelium [52]. Since HMGA1 has functions that likely affect regeneration [3, 9, 10] we asked: is the level of HMGA1 expression in BC associated with the regeneration capacity of BC? Primary BC from human SAE of 17 of nonsmokers, 14 healthy smokers and 16 COPD smokers described by Staudt *et al*. [52] were assessed for the capacity of the purified BC to differentiate in ALI culture. Based on HMGA1 expression in the SAE BC at the start of the ALI cultures, samples were categorized as top HMGA1 expressors (HMGA1 expression highest quartile), and bottom HMGA1 expressors (HMGA1 expression lowest quartile; Figure 2A). The capacity of these cells to regenerate airway epithelium in ALI was assessed using Kaplan-Meier survival analysis comparing HMGA1 top expressors and bottom expressors. HMGA1 BC top expressors had a better capacity to regenerate airway epithelium compared to HMGA1 BC bottom expressors which had decreased regeneration capacity (*p* = 0.03, log-rank test; Figure 2B).

Based on the data demonstrating an association between the level of HMGA1 expression in BC and airway epithelial regeneration, subsequent studies were designed to explore the role of HMGA1 in differentiation of normal human airway epithelium. siRNA was used to suppress HMGA1 expression in normal airway BC and the BC were then assessed for the capacity to form spheres in 3D matrigel culture, differentiate on ALI on type IV collagen, form an intact barrier, and repair following injury.

### Sphere formation, proliferation and differentiation

**Figure 3:**
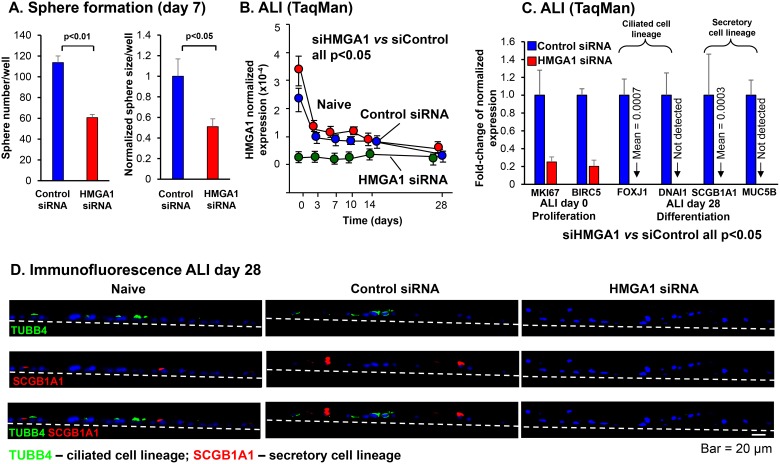
Effect of silencing HMGA1 on BC proliferation, and ciliated and secretory differentiation gene expression (**A**) Sphere formation. LAE BC were initially cultured using culture method “BC-1” and then cultured in matrigel following transfection at day 0 with HMGA1 siRNA compared to control siRNA. Shown is sphere number (*n* = 3 wells assessed) and sphere size represented by the area of the sphere in the images (*n* = 3 wells counted, 4 images per well, 12 images total) at day 7. Sphere size in control siRNA transfected samples was normalized to 1. (**B**) HMGA1 expression in BC placed on ALI following transfection with HMGA1 siRNA, control siRNA or no transfection (naive). HMGA1 expression was quantified by TaqMan. At all time points, control siRNA *vs* HMGA1 siRNA, *n* = 5, each group; *p <* 0.05. LAE BC from Lonza; see Methods, BC-1 and ALI-1. (**C**) Effect of HMGA1 silencing on expression of proliferation and differentiation-related genes. BC were transfected with HMGA1 siRNA or control siRNA at day 0. BC were cultured on ALI and assessment was carried out at day 0 or day 28. Quantification was by TaqMan. Shown is proliferation-related marker of proliferation Ki-67 (MKI67) and proliferation/survival-related baculoviral IAP repeat containing 5 (BIRC5), ciliated cell markers forkhead box J1 (FOXJ1), dynein axonemal intermediate chain 1 (DNAI1), and secretory cell markers secretoglobin family 1A member 1 (SCGB1A1), mucin 5B, oligomeric mucus/gel-forming (MUC5B). Expression was normalized to 18S rRNA, with gene expression in control siRNA transfected samples normalized to 1. *n* = 5, each group. LAE BC from Lonza; see Methods, BC-1 and ALI-1. (**D**) Immunofluorescence assessment of the effect of HMGA1 silencing in BC on expression of the ciliated cell marker β-tubulin IV (TUBB4) and secretory cell marker SCGB1A1. SAE from healthy nonsmokers were sampled by bronchoscopic brushing. Purified BC were transfected by control and HMGA1 siRNAs and the capacity of these cells to differentiate assessed on ALI culture; see Methods, BC-2 and ALI-2. At ALI day 28, the membranes were paraffin embedded and sectioned. The cells grown on the membranes were then characterized by immunofluorescences using ciliated cell marker TUBB4 (green) and secretory cell marker SCGB1A1 (red). Dashed line represents the ALI membrane.

Formation of tissue spheres in three dimensional culture is a common *in vitro* measure of progenitor function. To assess BC ability to form spheres in matrigel, airway BC were transfected with siRNA directed against HMGA1 or control siRNA and embedded in matrigel. At day 7, both sphere number (*p <* 0.01) and sphere size (*p <* 0.001) was reduced in HMGA1-silenced samples, suggesting reduced proliferation capacity in airway BC with low HMGA1 expression (Figure 3A, Supplementary Figure 2). To further specify the role of HMGA1 on the proliferation of BC and exclude the influences of other cell types in matrigel or on ALI assays, we performed the HMGA1 siRNA transfection in the expanding BC, and assessed the expression of proliferation marker MKI67 by TaqMan and compared the cell number of control and HMGA1-silenced BC at the end of the BC culture. Consistent with the findings in matrigel and ALI cultures, MKI67 expression in BC is down-regulated 24, 48 and 72 hr after HMGA1 siRNA transfection, suggesting lower proliferation capacity in HMGA1-silenced BC (siHMGA1 *vs* siControl, all *p <* 0.05; Supplementary Figure 3A). The cell number was counted 72 hr after transfection. HMGA1 silencing significantly reduces cells numbers of growing BC, supporting the concept that BC with suppressed HMGA1 expression have impaired proliferation capacity (siHMGA1 *vs* siControl, *p <* 0.01; Supplementary Figure 3B).

Consistent with the ALI data demonstrating BC with high HMGA1 expression had a greater capacity to differentiate compared to BC with low HMGA1 expression (Figure 2), when HMGA1 was suppressed using the same siRNA strategy and the BC placed on ALI culture (Figure 3B), the expression of proliferation genes (MKI67, BIRC5), ciliated cell-related differentiation genes (FOXJ1, DNAI1) and secretory cell-related genes (SCGB1A1, MUC5B) were all suppressed compared with culture treated with control siRNA (Figure 3C). Consistent with this data, suppression of HMGA1 expression in BC resulted in the suppressed expression of ciliated (TUBB4)- and secretory (SCGB1A1)-related genes at the protein level (Figure 3D).

### Barrier integrity of the differentiated airway epithelium

**Figure 4:**
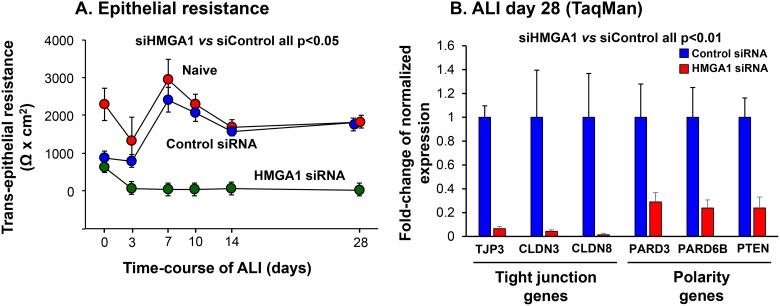
Consequences of HMGA1 down-regulation on airway epithelial barrier formation and barrier-related gene expression BC were transfected with HMGA1 or control siRNA before being placed on ALI culture. Assessment was carried over 28 days. (**A**) Transepithelial resistance. *n* = 5, each group. (**B**) Expression of tight junction and polarity-related genes. Shown is data at day 28 assessed by TaqMan for tight junction protein 3 (TJP3), claudin 3 (CLDN3), claudin 8 (CLDN8), par-3 family cell polarity regulator (PARD3), par-6 family cell polarity regulator β (PARD6B) and phosphatase and tensin homolog (PTEN). Data was normalized to 18S rRNA. Gene expression in control siRNA transfected samples was normalized to 1. *n* = 5, each group. For A and B, LAE BC from Lonza, see Methods, BC-1 and ALI-1.

An important function of airway epithelium is to form a physical barrier which segregates the external environment to maintain airway homeostasis. The role of HMGA1 in maintaining airway barrier integrity was assessed by measuring the transepithelial resistance (TER) of the airway epithelium derived from HMGA1 siRNA- or control siRNA-transfected BC cultured in ALI. At all time points, suppression of HMGA1 expression significantly impaired TER (Figure 4A, siHMGA1 *vs* siControl, all *p <* 0.05). Based on the knowledge that effective formation of the airway barrier relies on the expression of tight junction and cell polarity-related genes [53], we assessed the expression of this class of genes. Consistent with the TER data, siRNA-mediated HMGA1 silencing was associated with suppression of expression of tight junction-related genes TJP3, CLDN3 and CLDN8 and cell polarity-related genes PARD3, PARD6B and PTEN (Figure 4B, all *p <* 0.01).

### Wound closure of airway epithelium following injury

**Figure 5:**
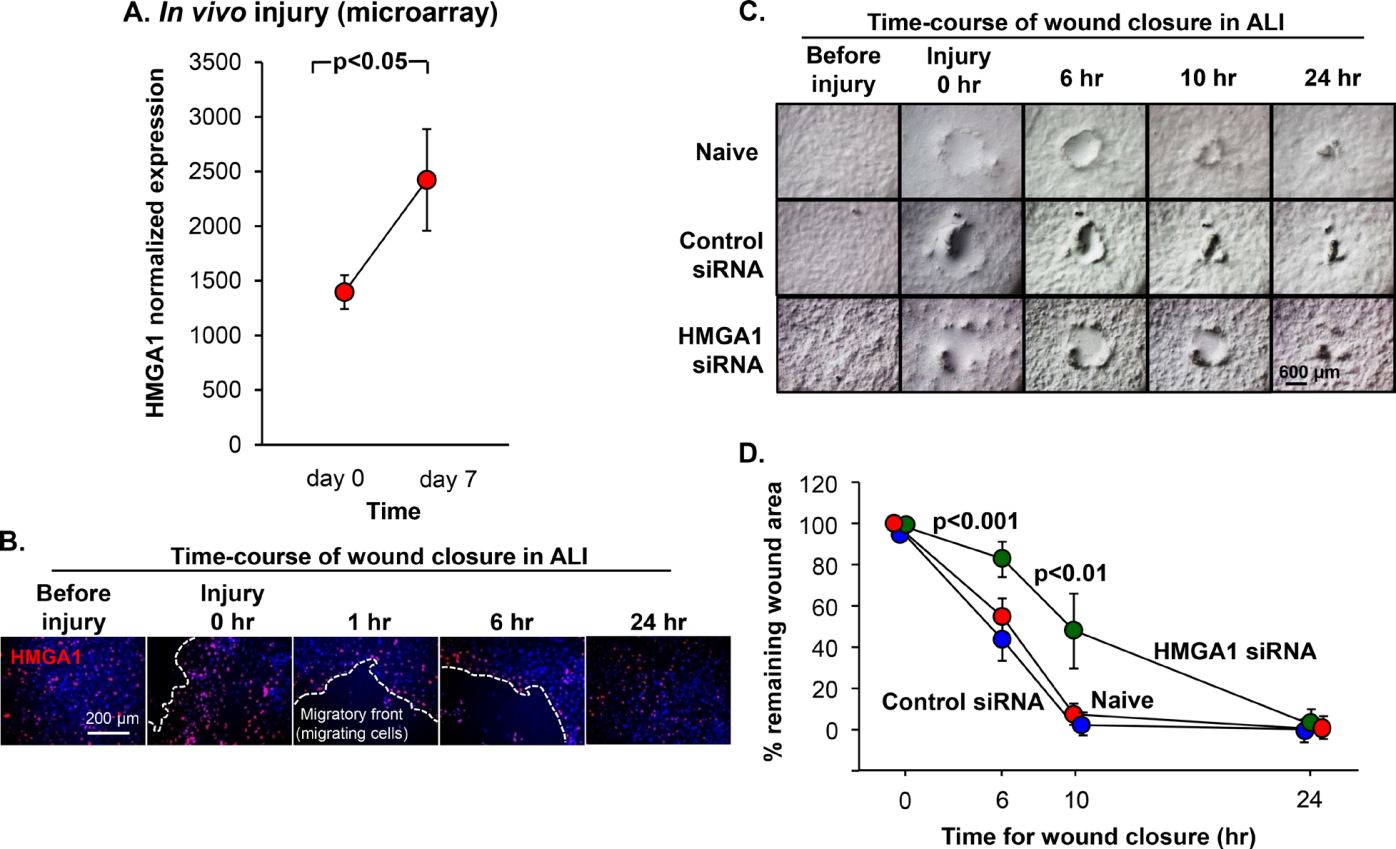
Role of HMGA1 in BC-mediated airway epithelial wound repair (**A**) The epithelium of the large (2^nd^ - 3^rd^ order) bronchi of 3 nonsmokers and 4 smokers was sampled at rest and then the same individuals were resampled following mechanical (brush) injury. All seven individuals were assessed at rest (day 0), and resampled at day 7 following injury. HMGA1 expression was assessed by Affymetrix HG-U133 Plus 2.0 array. (**B**) LAE BC purified from brushing were cultured by Methods BC-1 and ALI-1. At ALI day 7, the basal/intermediate cell stage, cell layer was scratched using a 10 μl sterile pipette tip. Immunofluorescence assessment of top staining of HMGA1 (red) and DAPI (nuclei, blue) were performed at the indicated time points. (**C**) BC were transfected with HMGA1 or control siRNA or no siRNA (naive). At ALI day 7, the basal/intermediate cell stage, cell layer was scratched using a 10 μl sterile pipette tip. Wound closure was monitored at the indicated time points. LAE BC from Lonza, see Methods, BC-1 and ALI-1. (**D**) Wound area. Quantification was measured by ImageJ. The *p* values indicate the comparison between HMGA1 siRNA transfection *vs* negative control siRNA transfection, *n* = 6, each group.

Airway BC play a key role in the response to airway epithelial injury, re-establish the intact airway barrier, and finally regenerate functional airway epithelium [43, 44, 47, 54–58]. To explore the role of HMGA1 in the airway epithelium regeneration *in vivo*, we reassess the data of our previously published human airway epithelium *in vivo* injury study; we previously found that at day 7 after injury compared with resting epithelium, there were substantial differences in the gene expression pattern representing a “repair transcriptome” [59]. Interestingly, HMGA1 was upregulated at day 7 compared with day 0, consistent with a role for HMGA1 in airway regeneration *in vivo* (Figure 5A). In agreement with these findings, a wound closure assay was performed by scratching at the basal/intermediate stage ALI day 7, and showed that HMGA1-expressing BC accumulated at the repairing wound edge (Figure 5B). To assess the function of HMGA1 in airway wound repair, wound repair was assessed *in vitro* following delivery of siRNA directed toward HMGA1. BC with down-regulated HMGA1 or control BC were cultured on ALI and an injury was made on the epithelium at ALI day 7. The data demonstrates that the early phases of the wound closure process were significantly suppressed in the HMGA1 silenced BC (Figure 5C, 5D).

### Expression of genes associated with airway remodeling in COPD

**Figure 6:**
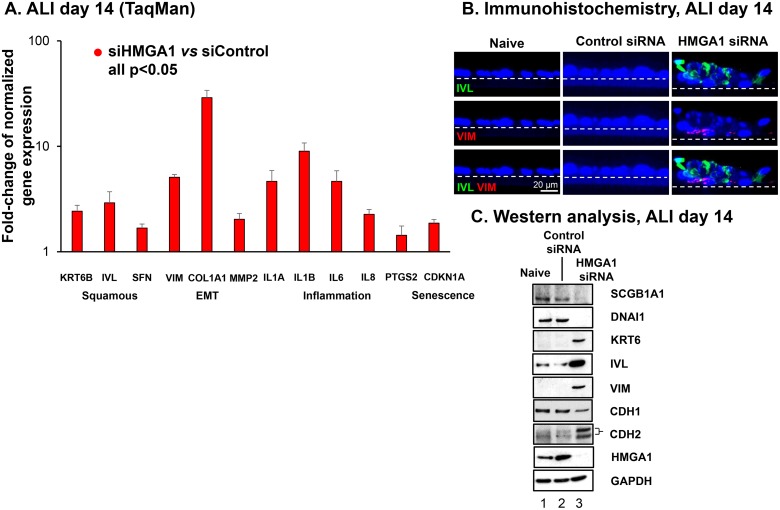
Consequences of HMGA1 silencing in airway BC on the expression of genes associated with airway remodeling in COPD (**A**) Effect of HMGA1 silencing on expression of squamous, epithelial-mesenchymal transition (EMT), inflammation and senescence-related genes in BC differentiating on ALI. Gene expression was assessed by TaqMan at ALI day 14. Shown are squamous-related genes keratin 6B (KRT6B), involucrin (IVL) and stratifin (SFN); EMT-related genes vimentin (VIM), collagen type I α1 (COL1A1), and matrix metallopeptidase 2 (MMP2); and inflammation-related genes interleukin 1α (IL1A), interleukin 1β (IL1B), interleukin 6 (IL6), interleukin 8 (IL8), prostaglandin-endoperoxide synthase 2 (cyclooxygenase 2 or PTGS2) and senescence-related gene cyclin dependent kinase inhibitor 1A (CDKN1A). Data was normalized to 18S rRNA. Gene expression in control siRNA transfected samples was normalized to 1. *n* = 6, each group. For A and B, LAE BC from Lonza, see Methods, BC-1 and ALI-1. (**B**) Immunofluorescence assessment of protein expression. Purified normal SAE BC were transfected by control and HMGA1 siRNAs and the capacity of these cells to differentiate were assessed in ALI culture. At ALI day 14, the membranes were paraffin embedded and sectioned. The cells grown on the membranes were then characterized by immunofluorescences using squamous cell marker IVL (green) and EMT marker VIM (red). Dashed line indicates the ALI membrane. See Methods, BC-2 and ALI-2. (**C**) Western analysis. HMGA1 was silenced by HMGA1 siRNA transfection in BC. At ALI day 14, protein was harvested and Western analysis was performed using SCGB1A1, DNAI1, keratin 6 (KRT6), IVL, VIM, cadherin 1 (CDH1, also known as E-cadherin), cadherin 2 (CDH2, also known as N-cadherin), and HMGA1 antibodies, respectively. GAPDH was used as a loading control. LAE BC from Lonza, see Methods, BC-1 and ALI-1.

Based on the foregoing data demonstrating that reduced expression of HMGA1 inhibits normal differentiation of airway epithelium BC, and given prior observations that dysregulation of BC function can lead to production of cells with a squamous character, we hypothesized that low expression of HMGA1 would correlate with the expression of common markers of squamous epithelial cells [51, 60, 61]. To evaluate this hypothesis, we assessed genes associated with airway remodeling in COPD by comparing gene expression of epithelium derived from HMGA1 siRNA-transfected BC and BC transfected with control siRNA. Interestingly, the BC with silenced HMGA1 exhibited up-regulated expression, not only of the squamous-related genes KRT6B, IVL and SFN, but also EMT-related genes VIM, COL1A1, and MMP2, and inflammation-related genes IL1A, IL1B, IL6, IL8 and PTGS2 (Figure 6A, siHMGA1 *vs* siControl all *p <* 0.05) [61, 62]. Of note, the expression of senescence marker CDKN1A (also named p21) was up-regulated in HMGA1-silenced airway epithelium (Figure 6A). Consistent with the mRNA data, immunofluorescence assessment of ALI at day 14 and Western analysis of ALI at day 14 showed up-regulation of squamous and EMT related proteins (KRT6, IVL and VIM, CDH2) but down-regulation of normal differentiation proteins (SCGB1A1, DNAI1) and epithelial marker gene (CDH1; Figure 6B, 6C). In addition, reduced expression of epithelial-specific tight junction and polarity-related genes on ALI indicates the appearance of EMT-like phenotype (Figure 4B).

## DISCUSSION

The basal stem/progenitor cells of the human airway epithelium are central to the replenishment of the airway epithelium associated with normal cell turnover and following airway epithelial injury [43, 44, 47, 54–58]. The data in our study demonstrates that HMGA1 not only plays a critical role in permitting normal airway differentiation and repair following injury, but in the absence of high levels of HMGA1 expression, the BC resort to a default, pathologic, COPD-like phenotype and can no longer function to repair the injured epithelium in a normal fashion (Supplementary Figure 4).

“Architectural transcription factors” such as HMGA1 function through modulating the conformation of DNA, forming suitable architecture to allow transcriptional machinery to function [3, 9, 10, 63, 64]. HMGA1, a prototype architectural transcription factor, is expressed in undifferentiated and rapid proliferating cells, but HMGA1 expression is typically low or undetectable in fully differentiated cells [3]. Through protein-DNA interactions, HMGA1 alters chromatin structure, and forms high-order stereospecific complexes on the promoter/enhancer regions of target genes, functioning to regulate gene expression [3, 65]. HMGA1 can also function through protein-protein interactions independent of binding to DNA [3], and plays a role in chromatin remodeling of promoters [66].

Our study focused on humans only. The mouse small airway epithelium has a very different cell population than humans; in humans, the BC is the progenitor cell, while in mouse small airway epithelium the club cells are the progenitor cells. A subpopulation of basal cells has been identified in the pseudostratified epithelium of upper airways of the mouse [57, 67, 68]; these investigators have demonstrated the potential of BC in the upper airways to regenerate airway epithelium in response to injury *in vivo* [67].

Relevant to the function of HMGA1, serial passaging of human BC leads to a senescence state characterized by growth arrest accompanied by diminished differentiation capacity [69, 70]. Silencing of HMGA1 by siRNA decreases normal BC proliferation and differentiation and may play a role in BC senescence. Consistent with this concept, we identified up-regulated expression of proinflammatory genes and matrix metalloproteinases (MMPs) in HMGA1-down-regulated epithelium in ALI. Elevated expression of proinflammatory cytokines and chemokines and MMPs is referred to as a “senescence-associated” secretory phenotype, one of the key characteristics in distinguishing senescent cells with other proliferation-arrested cells [71]. To further validate the role of HMGA1 in senescence-prevention of BC, we knocked down HMGA1 in ALI and assessed the expression of the senescence marker CDKN1A. Silencing HMGA1 in ALI significantly up-regulated CDKN1A gene expression indicating the induction of senescence. In the context of these observations, we propose that HMGA1 mediates BC differentiation through prevention of BC senescence, and therefore, maintaining BC homeostasis.

HMGA1 plays key roles in BC differentiation and regeneration. However, due to its nature as an architectural transcription factor, the way that HMGA1 functions is, most likely, permissive, i.e., HMGA1 coordinates with DNA, RNA and proteins to assist their normal functions. When HMGA1 expression is reduced by siRNA transfection, the HMGA1 effectors cannot work appropriately resulting in COPD-related pathologic changes. Cigarette-smoke induces fundamental gene expression and epigenetic changes associated with the capacity of BC to differentiate and regenerate [44–46, 52, 60, 72]. In this case, it is not a surprise that many HMGA1 effectors will be dysregulated in BC from COPD smokers. While restoration of HMGA1 in BC from COPD smokers may at first be an attractive therapeutic concept, previous studies have shown that the over-expression of HMGA1 is associated with cancer [3], suggesting there may be unfavorable off-target effects of HMGA1 over-expression in BC.

EMT has been implicated in the wound healing. Even though HMGA1 siRNA-treated cells express mesenchymal markers such as VIM, COL1A1 and MMP2, they are a bit slow to appear during the wound closure. Because EMT is a complex process including extensive gene expression, cellular morphology and phenotype changes of the epithelial cells [73, 74], dysregulation of architectural transcription factor HMGA1 may alter the time course of regular EMT, and eventually delayed the wound repair function of BC. It has been reported that HMGA1 plays anti-apoptotic role by modulating BCL2 in human breast carcinoma cells [75]. Apoptosis may occur in HMGA1-silenced cells during the wound healing. This could partially explain the inability of HMGA1-silenced cells to close the wound.

Low expression levels of HMGA1 shift BC to an abnormal differentiation pathway, and thus the association between HMGA1 expression and known risk factors or clinical phenotypes for COPD is of interest. In the context of cigarette smoking, HMGA1 may be associated with lung function. Further study with large population will be helpful to assess this hypothesis. We have previously investigated the cigarette smoke-mediated changes of airway epithelium by assessing normal human airway BC differentiation in ALI cultures in the presence of nontoxic concentrations of cigarette smoke extract (CSE) [76]. In that study, we found CSE suppresses ciliated cell and secretory cell differentiation but increases squamous cell differentiation consistent with the observation of silencing of HMGA1 in the current study. These data suggest that HMGA1 may play a key role in cigarette smoke-induced changes in the human airway epithelium.

It is clear from the data that down-regulation of HMGA1 is associated with suppression of expression of genes critical to normal differentiation and up-regulation of genes linked to abnormal differentiation relevant to smoking and COPD. Future use of systemic anti-HMGA1 strategies should be cognizant of potential effects of reduced HMGA1 activity in normal epithelia, and, if necessary, adopt methods for targeting HMGA1 therapies specifically to tumor cells.

## METHODS

### Clinical samples and RNA sequencing

Human large airway epithelium (LAE, 3rd-4th order bronchi) of healthy nonsmokers, healthy smokers and smokers with COPD, and small airway epithelium (SAE, 10th-12th order) of healthy nonsmokers were obtained by fiberoptic bronchoscopy and brushings. Subjects were recruited under a protocol approved by the Weill Cornell Medical College Institutional Review Board, with written informed consent. Details regarding inclusion/exclusion criteria for the clinical phenotypes are described in Supplementary Methods. The “n” for each group is detailed with the data. RNA purification, quality control and sequencing [Illumina HiSeq 2500 (2×125 bp)] of the clinical samples and BC culturing were as per protocol (Illumina, San Diego, CA) as previously described [77, 78] (see Supplementary Methods). The paired-end reads were processed with STAR (2.3.1z13_r470) to align reads to the GRCh37/hg19 human reference genome and RefSeq gene definitions (version of 2014-06-02) [79]. Gene expression quantification was performed using Cufflinks (2.2) against the RefSeq gene definitions [80]. Non-aligned reads were segmented using STAR and re-aligned, thereby aligning reads that span introns and determining junction splice sites. Cufflinks assembled reads into transcripts and assembled reads were then merged using Cuffmerge. To correct for transcript length and coverage depth, raw paired-end reads were converted into fragments per kilobase of exon per million fragments sequenced (FPKM). Reads generated were directly proportional to transcript relative abundance. The raw data and FPKM values are publically available at the Gene Expression Omnibus (GEO) site (http://www.ncbi.nlm.nih.gov/geo/), accession number GSE86064.

### Basal cells

SAE BC were isolated from SAE brushings as previously described [52]. Depending on the specific experiment as specified in the figure legends, the SAE BC were cultured as follows: (1) “BC-1” – on plastic in bronchial epithelial growth media (BEGM, Lonza, Basel, Switzerland); antibiotics supplied by the manufacturer were replaced with gentamicin (50 μg/ml; Sigma, St Louis, MO), amphotericin B (1.25 μg/ml; Life Technologies, Carlsbad, CA), and penicillin-streptomycin (50 units/ml penicillin, 50 μg/ml streptomycin; Life Technologies); or (2) “BC-2” – on type IV collagen in small airway epithelial cell growth medium (SAGM, Lonza) to expand the cells, followed by culture on plastic in S-ALI growth medium (Lonza). LAE BC were either from Lonza or isolated from LAE brushings as previously described [47], and cultured using the “BC-1” culture conditions. For both the SAE and LAE BC cultures, all studies were limited to culture passages 0 to 3 and all studies were carried out at day 7 to 8 of culture, when the cells were 70% to 80% confluent.

### Air-liquid interface cultures

BC were differentiated using an air-liquid interface (ALI) model [47–49]. Briefly, at ALI day −2, BC were seeded at a density of 1.5–2 × 10^5^ cells/well onto a 0.4 μm pore-sized 24 well transwell filters (Corning Incorporated, Corning, NY) pre-coated with type IV collagen (Sigma, St. Louis, MO). At ALI day 0, the medium on the apical side was removed and the cells were exposed to air to facilitate the differentiation. Depending on the specific experiment as specified in the figure legends, the cells on ALI were cultured as follows: (1) “ALI-1” - the ALI were initially cultured in a 1:1 mixture of DMEM and Ham's F12 medium supplemented with 5% defined fetal bovine serum (GE Healthcare Life Sciences, Pittsburgh, PA) and 50 μg/ml of gentamicin, 1.25 μg/ml of amphotericin B, and penicillin-streptomycin (50 units/ml of penicillin and 50 μg/ml of streptomycin) for one day. At ALI day −1, the medium was changed to a 1:1 mixture of DMEM and Ham's F12 supplemented with 2% Ultroser G (BioSerpa S.A., Cergy-Saint-Christophe, France) and antibiotics as described above. At ALI day 0, the media on the apical side were removed and the cells were exposed to air to facilitate the differentiation with Ultroser G-based media supplied from the basolateral side. The cells were grown at 37° C, 8% CO_2_ from ALI day −2 to day 5. Following ALI day 5, the cells were grown at 37° C, 5% CO_2_ until harvested; or (2) “ALI-2” - the ALI were cultured at 37° C, 5% CO_2_ in S-ALI growth medium from day −2 to day 0, and S-ALI differentiation medium (Lonza) from ALI day 0 to harvest. The medium was changed every other day from the basolateral side.

For suppressing HMGA1 gene expression in ALI, 1.5–2 × 10^5^ cells/well BC were transfected with the HMGA1 or control siRNA (5 pmol, # 4427037 siRNA ID s6667 for HMGA1 siRNA, # 4390843 for control siRNA, Life Technologies, Carlsbad, CA) using the lipofectamine RNAiMAX reagent (Life Technologies) at ALI day −2. Transfection mixtures were mixed with BC suspension and seeded onto 24 well transwells. At ALI day −1, the medium was changed and then the cells were cultured based on the standard ALI protocol. For suppressing HMGA1 gene expression in expanding BC, the transfection was performed using lipofectamine RNAiMAX reagent according to the manufacturer's protocol.

### Consequence of high and low BC HMGA1 expression on the ability of BC to successfully differentiate

We have previously demonstrated that SAE BC from COPD smokers and healthy smokers have a decreased capacity to differentiate on ALI [52]. In that study, primary BC purified from human SAE sampled by bronchoscopic brushing of 47 individuals (17 nonsmokers, 14 healthy smokers and 16 COPD smokers) were plated on ALI and cultured for 28 days. On ALI day 0, RNA was harvested. The ability of BC to form a differentiated epithelium was assessed over the course of 28 days. Cultures that failed to maintain differentiation to day 28 on ALI exhibited spontaneous generation of holes in the cellular monolayer, followed by collapse and failure of the culture at 10 to 28 days. The survival time (days) on ALI for each BC from different subjects was recorded. To determine if the level of expression of HMGA1 in the SAE BC correlated with the survival time of ALI cultures, in the present study we reassessed the same samples at the start of the cultures (day 0, before differentiation on ALI) for HMGA1 expression using TaqMan PCR. SAE BC from the day 0 ALI cultures were divided by quartile with highest 12 expressors categorized as “top HMGA1” expressors (HMGA1 expression highest quartile, *n* = 12), and the lowest 12 expressors categorized as “bottom HMGA1” expressors (HMGA1 expression lowest quartile, *n* = 12). Using the survival data from the prior study [52], Kaplan-Meier survival analysis and the log-rank test were used to compare the successful differentiation of HMGA1-top and -bottom quartile expressors.

### BC sphere formation

To assess the role of HMGA1 in the ability of BC to proliferate in matrigel, 24 well transwell filters were pre-coated with 50% matrigel (basement membrane matrix growth factor reduced, Corning Incorporated). The BC were mixed with matrigel (5%) and the suspension was seeded onto 24 well transwell filters. Ultroser G media (2%) was changed every other day from the basolateral side. At day 7 of the matrigel culture, the total sphere number in 3 wells for each group was counted visually by microscopy, and sphere size represented by the area of the sphere in 12 images from 3 wells (4 images per well) for each group was measured using ImageJ. To evaluate the consequences of HMGA1 knock down, 200 BC were transfected prior to culture with 5 pmol HMGA1 or control siRNA using lipofectamine RNAiMAX reagent as described above.

### Gene expression

TaqMan PCR was used for assessment of gene expression in the ALI cultures. ALI cultures were harvested in TRIzol (Invitrogen, Carlsbad, CA), followed by RNA extraction by RNeasy MinElute Cleanup Kit (Qiagen, Valencia, CA), and reverse transcription. TaqMan PCR analyses were performed using 7500 Real Time PCR System, Sequence Detection Software version 1.4 (Applied Biosystems, Foster City, CA), and commercially available TaqMan probes (Life Technologies) with standard protocols. The genes assessed were HMGA1, marker of proliferation Ki-67 (MKI67), baculoviral IAP repeat containing 5 (BIRC5), forkhead box J1 (FOXJ1), dynein axonemal intermediate chain 1 (DNAI1), secretoglobin family 1A member 1 (SCGB1A1), mucin 5B, oligomeric mucus/gel-forming (MUC5B), junction protein 3 (TJP3), claudin 3 (CLDN3), claudin 8 (CLDN8), par-3 family cell polarity regulator (PARD3), par-6 family cell polarity regulator β (PARD6B), phosphatase and tensin homolog (PTEN), keratin 6B (KRT6B), involucrin (IVL), stratifin (SFN), vimentin (VIM), collagen type I α1 (COL1A1), matrix metallopeptidase 2 (MMP2), interleukin 1α (IL1A), interleukin 1β (IL1B), interleukin 6 (IL6), interleukin 8 (IL8), prostaglandin-endoperoxide synthase 2 (cyclooxygenase 2 or PTGS2), and cyclin dependent kinase inhibitor 1A (CDKN1A; see Supplementary Table 1 for details).

### Immunohistochemistry and immunofluorescence assessment of gene expression

Immunohistochemistry and immunofluorescence gene expression of lung biopsy samples, ALI cultures and cytopreparations were performed using protocol described previously [61, 81]. The following primary antibodies were used: HMGA1 (ab4078, 1:3000, Abcam, Cambridge, UK), SCGB1A1 (RD181022220, 1:100, BioVendor, Asheville, NC), β-tubulin IV (TUBB4, MU-178-UC, 1:100, Biogenex, Fremont, CA), IVL (MS-126-P, 1:100, Neomarkers, Fremont, CA), VIM (ab133260, 1:100, Abcam), keratin 5 (KRT5, PA1-37974, 1:100, Thermo Scientific, Rockford, IL) and mucin 5AC, oligomeric mucus/gel-forming (MUC5AC, VP-M567, 1:100, Vector, Burlingame, CA). Immunohistochemistry images were acquired using a Nikon Microphot-SA microscope (Nikon, Melville, NY) with Olympus DP70 CCD camera (Olympus, Center Valley, PA). Immunofluorescence images were captured with a Zeiss Axioplan body microscope and a Zeiss high resolution monochrome camera, and analyzed by AxioVision Rel 4.8 software (Carl Zeiss, Jena, Germany).

### Epithelial barrier

The BC-derived airway epithelial barrier integrity in ALI was assessed by measuring the transepithelial resistance (TER; Ω x cm^2^; Millicell-ERS epithelial ohmmeter; Millipore, Bedford, MA) [53]. At ALI day 28, expression of tight junction and polarity-related genes including TJP3, CLDN3, CLDN8, PARD3, PARD6B and PTEN were assessed by TaqMan (see Supplementary Table 1 for details).

### *In vivo* injury model

We have previously evaluated the response of the human airway epithelium transcriptome to *in vivo* injury [59]. In that study, we used bronchoscopy and brushing to denude the airway epithelium of healthy individuals, sequentially sampled the same region 7 days later, and assessed gene expression by Affymetrix HG-U133 Plus 2.0 array. The injured area was completely covered by a partially redifferentiated epithelial layer after 7 days. At day 7 compared with resting epithelium at day 0, there were substantial differences in gene expression pattern representing a “repair transcriptome.” For the present study, we re-analyzed the dataset for HMGA1 gene expression at day 0 and day 7 following *in vivo* injury.

### Culture wound model

A wound closure assay was used to assess the capacity of naive BC and BC with suppressed HMGA1 expression to initiate wound repair [82, 83]. At ALI day 7 of basal/intermediate cell stage, the cell layer was scratched using a 10 μl sterile pipette tip. The wound area was recorded at time points indicated in the figure legends of the specific experiments using an Olympus IX71 microscope with Olympus DP73 camera and quantified using ImageJ. Pre-treatment of the BC with HMGA1 and control siRNA was as described above.

### Western analysis

To assess protein expression in the BC ± HMGA1 siRNA suppression, cells from ALI day 14 were lysed in RIPA buffer (Thermo Scientific) containing protease/phosphatase inhibitor cocktail (Cell Signaling Technology, Danvers, MA). Equal amounts of protein samples were boiled for 5 min and run on a NuPAGE 4-12% Bis-Tris mini gel (Invitrogen, Carlsbad, CA). Subsequently, proteins were transferred onto a PVDF membrane (Invitrogen). The membranes were blocked at 23° C in 5% nonfat milk (Santa Cruz Biotechnology, Santa Cruz, CA) in PBS containing 0.1% Tween-20 (PBST). The membrane was then incubated with specific antibodies in blocking buffer 4° C overnight, washed 3 times for 5 min each with PBST and incubated with an anti-rabbit or anti-mouse antibody conjugated to horseradish peroxidase (GE Healthcare Life Sciences, Pittsburgh, PA) in blocking buffer (23° C, 1 hr). The membranes were washed again 3 times for 5 min each with PBST, and the proteins were visualized by adding ECL prime Western blotting detection reagents (GE Healthcare Biosciences, Pittsburgh, PA) and exposure to an X-ray film. The primary antibodies included: HMGA1 (ab4078, 1:6000, Abcam), SCGB1A1 (RD181022220, 1:2000, BioVendor), DNAI1 (HPA021649-100UL, 1:2000, Sigma), keratin 6 (KRT6, LS-C88463, 1:2000, LifeSpan Biosciences, Seattle, WA), IVL (MS-126-P, 1:2000, Neomarkers), VIM (ab133260, 1:2000, Abcam), cadherin 1 (CDH1, also known as E-cadherin, #3195, 1:2000, Cell Signaling Technology), cadherin 2 (CDH2, also known as N-cadherin, #14215, 1:2000, Cell Signaling Technology), and GAPDH (sc-47724, 1:2000, Santa Cruz Biotechnology).

### Statistical analysis

A two-tailed Student's *t*-test was used for statistical analyses. In all analyses, a *p <* 0.05 was deemed significant, unless specified.

## SUPPLEMENTARY MATERIALS FIGURES AND TABLES


