# Supplementary material for: Mandatory role of HMGA1 in human airway epithelial normal differentiation and post-injury regeneration
Source: Oncotarget. 2018 Feb 16;9(18):14324–37. doi: 10.18632/oncotarget.24511 (PMC5865673; doi:10.18632/oncotarget.24511)
Supplement: Supplementary file 1 [file oncotarget-09-14324-s001.pdf]

Mandatory role of HMGA1 in human airway epithelial normal differentiation and post-injury regeneration

SUPPLEMENTARY MATERIALS

Inclusion and exclusion criteria for clinical phenotypes nonsmokers

Inclusion criteria

- Must be capable of providing informed consent
- Males and females, age 18 or older
- Females - not pregnant
- Never-smokers by history, with current smoking status validated by the undetectable levels of following metabolites: urine nicotine <2 ng/ml and urine cotinine <5 ng/ml
- Good overall health without history of chronic lung disease, including asthma, and without recurrent or recent (within 3 months) acute pulmonary disease
- Normal physical examination
- Normal routine laboratory evaluation, including general hematologic studies, general serologic/ immunologic studies, general biochemical analyses, and urine analysis
- Negative HIV serology
- Normal FEV1 (≥80% predicted), FVC (≥80% predicted), FEV1/FVC (≥0.7 predicted) based on pre-bronchodilator spirometry, DLCO (≥80% predicted) and TLC (≥80% predicted)
- Normal estimated pulmonary artery pressure assessed by diameter of the main pulmonary artery ≤30 mm in chest CT scans
- Normal chest X-ray (PA and lateral)
- Normal electrocardiogram (sinus bradycardia, premature atrial contractions are permissible)
- Not taking any medications relevant to lung disease
- Willingness to participate in the study

Exclusion criteria

- Unable to meet the inclusion criteria
- Pregnancy
- Current active infection or acute illness of any kind
- Current alcohol or drug abuse
- Evidence of malignancy within the past 5 years
- Any evidence of interstitial lung disease, pulmonary hypertension, diastolic dysfunction or other disorders associated with a low DLCO
- Subjects with allergies to lidocaine

Healthy smokers

Inclusion criteria

- Must be capable of providing informed consent
- Males and females, age 18 or older
- Females - not pregnant
- Current daily smokers with pack-yr ≥5, validated by urine cotinine ≥104 ng/ml, a level based on our previous study of low level smoke exposure (Strulovici-Barel Y *et al.*, Am J Respir Crit Care Med 2010; 182:1524)
- Good overall health without history of chronic lung disease, including asthma, and without recurrent or recent (within 3 months) acute pulmonary disease
- Normal physical examination
- Normal routine laboratory evaluation, including general hematologic studies, general serologic/ immunologic studies, general biochemical analyses, and urine analysis
- Negative HIV serology
- Normal FEV1 (≥80% predicted), FVC (≥80% predicted), FEV1/FVC (≥0.7 predicted) basedon pre-bronchodilator spirometry, DLCO (≥80% predicted) and TLC (≥80% predicted)
- Normal estimated pulmonary artery pressure assessed by diameter of the main pulmonary artery ≤30 mm in chest CT scans
- chest X-ray (PA and lateral)
- Normal electrocardiogram (sinus bradycardia, premature atrial contractions are permissible)
- No medications relevant to lung disease
- Willingness to participate in the study

Exclusion criteria

- Unable to meet the inclusion criteria
- Pregnancy
- Current active infection or acute illness of any kind
- Current alcohol or drug abuse
- Evidence of malignancy within the past 5 years

- Any evidence of interstitial lung disease, pulmonary hypertension, diastolic dysfunction or other disorders associated with a low DLCO
- Subjects with allergies to lidocaine

COPD smokers

Inclusion criteria

- Must be capable of providing informed consent
- Males and females, age 18 or older
- Females - not pregnant
- Current daily smokers with pack-yr ≥5, validated by urine cotinine ≥104 ng/ml, a level based on our previous study of low level smoke exposure (Strulovici-Barel Y *et al.*, Am J Respir Crit Care Med 2010; 182:1524)
- Taking any or no pulmonary-related medication, including beta-agonists, anticholinergics, or inhaled corticosteroids
- Normal routine laboratory evaluation, including general hematologic studies, general serologic/immunologic studies, general biochemical analyses, and urine analysis
- Negative HIV serology and positive HIV serology
- Presence of COPD as defined by the GOLD criteria based on post-bronchodilator FEV1/FVC<0.7 (observed); stage I-IV but without evidence of respiratory failure
- Normal electrocardiogram (sinus bradycardia, premature atrial contractions are permissible)
- Normal estimated pulmonary artery pressure assessed by diameter of the main pulmonary artery ≤30 mm in chest CT scans
- Normal chest X-ray (PA and lateral)
- Willingness to participate in the study

Exclusion criteria

- Unable to meet the inclusion criteria
- Individuals in whom participation in the study would compromise the normal care and expected progression of their disease
- Current active infection or acute illness of any kind
- Current alcohol or drug abuse
- Evidence of malignancy within the past 5 years
- Any evidence of interstitial lung disease, pulmonary hypertension, diastolic dysfunction orother disorders associated with a low DLCO
- Individuals with asthma and with recurrent or recent (within three months) acute pulmonary infection
- Individuals with allergies to lidocaine

RNA purification and quality control for RNA sequencing

RNA extraction, cDNA synthesis and sequencing were performed as per protocol (Illumina, San Diego, CA). Briefly, total RNA from intact large and small airway epithelium and purified BC was extracted with TRIzol (Invitrogen, Carlsbad, CA) with subsequent RNA clean-up using the RNeasy MinElute RNA purification kit (Qiagen, Valencia, CA) to remove residual DNA. RNA integrity was assessed using the Agilent Technologies 2100 Bioanalyzer (Santa Clara, CA), and the RNA concentration was determined by the NanoDrop ND-1000 spectropho-tometer (NanoDrop Technologies, Wilmington, DE). Samples were stored in RNAsecure (Ambion, Austin, TX) at −80° C until further analysis. Total RNA was submitted to the New York Genome Center for RNA sequencing using Illumina HiSeq 2500 (2 × 125 bp). The TruSeq RNA Sample Prep Kit (Illumina, San Diego, CA) was used to generate poly(A)+ mRNA libraries from total RNA incorporating Sera-mag magnetic oligo(dT) beads. An RNA fragmentation kit (Illumina, San Diego, CA) was utilized for mRNA fragmentation. Random hexamer primers were next used to synthesize first and second strand cDNA. Klenow polymerase and T4 DNA polymerase were used to perform an “end repair” reaction to blunt the fragment ends, and 3′-5′ exonuclease was used to create the 3′ adenine overhanging tail, allowing ligation of amplification adapters. Ligation products were then separated on a 2% trisacetate EDTA-agarose gel for size selection, with subsequent purification using a QIA-quick gel extraction kit (Qiagen). The purified ligation products were PCR amplified with complementary primers, the resultant cDNA was purified with QIA-quick PCR purification kit (Qiagen, Valencia, CA), and its concentration was determined by the NanoDrop spectrophotometer.

Supplementary Table 1: TaqMan gene expression assay probes[1]

Gene symbol	TaqMan assay ID
HMGA1	Hs00852949_g1
MKI67	Hs01032443_m1
BIRC5	Hs04194392_s1
FOXJ1	Hs00230964_m1
DNAI1	Hs00201755_m1
SCGB1A1	Hs00171092_m1
MUC5B	Hs00861588_m1
TJP3	Hs00274276_m1
CLDN3	Hs00265816_s1
CLDN8	Hs00273282_s1
PARD3	Hs00969077_m1
PARD6B	Hs00325996_m1
PTEN	Hs02621230_s1
KRT6B	Hs00745492_s1
IVL	Hs00846307_s1
SFN	Hs00968567_s1
VIM	Hs00185584_m1
COL1A1	Hs00164004_m1
MMP2	Hs01548727_m1
IL1A	Hs00174092_m1
IL1B	Hs01555410_m1
IL6	Hs00985639_m1
IL8	Hs00174103_m1
PTGS2	Hs00153133_m1
CDKN1A	Hs00355782_m1

[1]From Life Technologies (Carlsbad, CA)

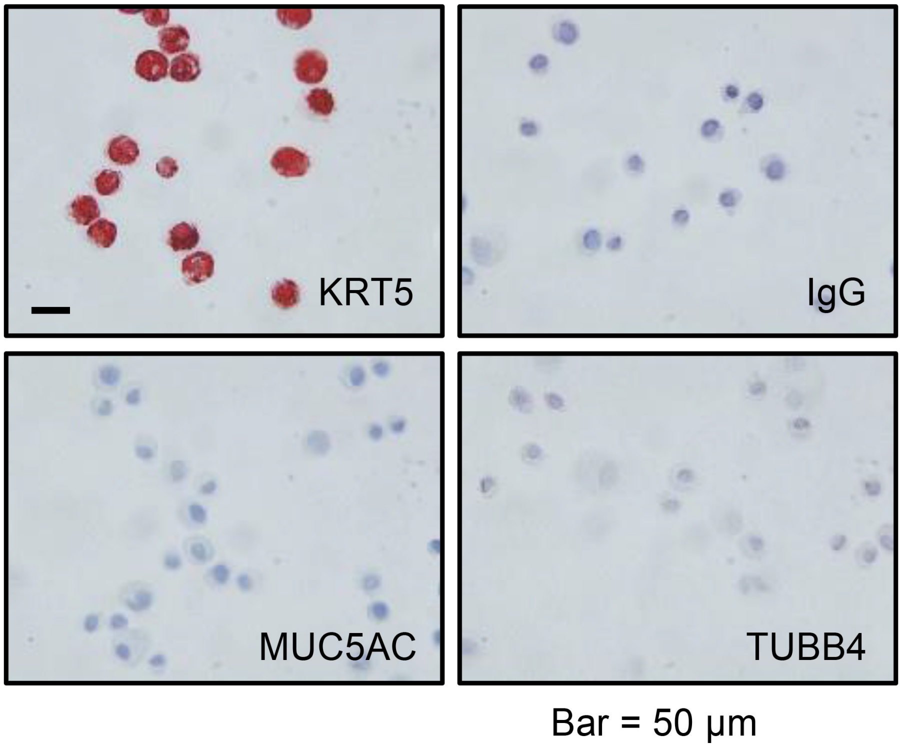

Supplementary Figure 1: Characterization of purified primary human airway BC. Immunohistochemical characterization of cytopreparations of primary human airway BC isolated using selective culture methods from small airway epithelial samples obtained by bronchoscopy from COPD smokers. Shown are keratin 5 (KRT5, BC), mucin 5AC, oligomeric mucus/gel-forming (MUC5AC, secretory cells) and TUBB4 (ciliated cells). IgG as a control. Cells were expanded on type IV collagen in small airway epithelial cell growth medium (SAGM, Lonza, Basel, Switzerland), followed by culture on plastic in bronchial epithelial growth media (BEGM, Lonza).

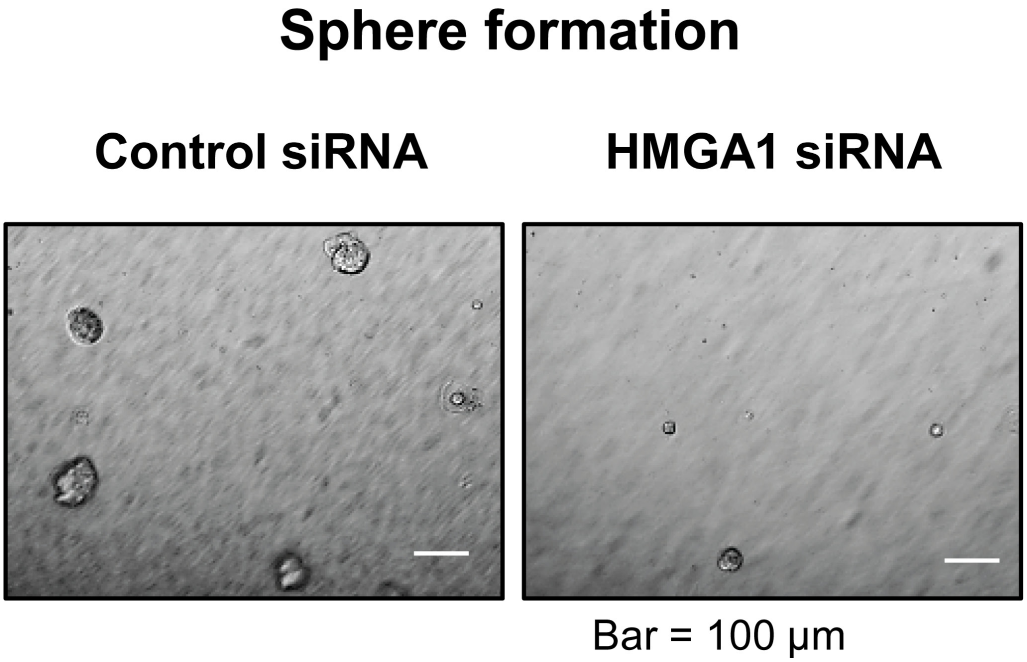

Supplementary Figure 2: Representative images corresponding to Figure 3A.

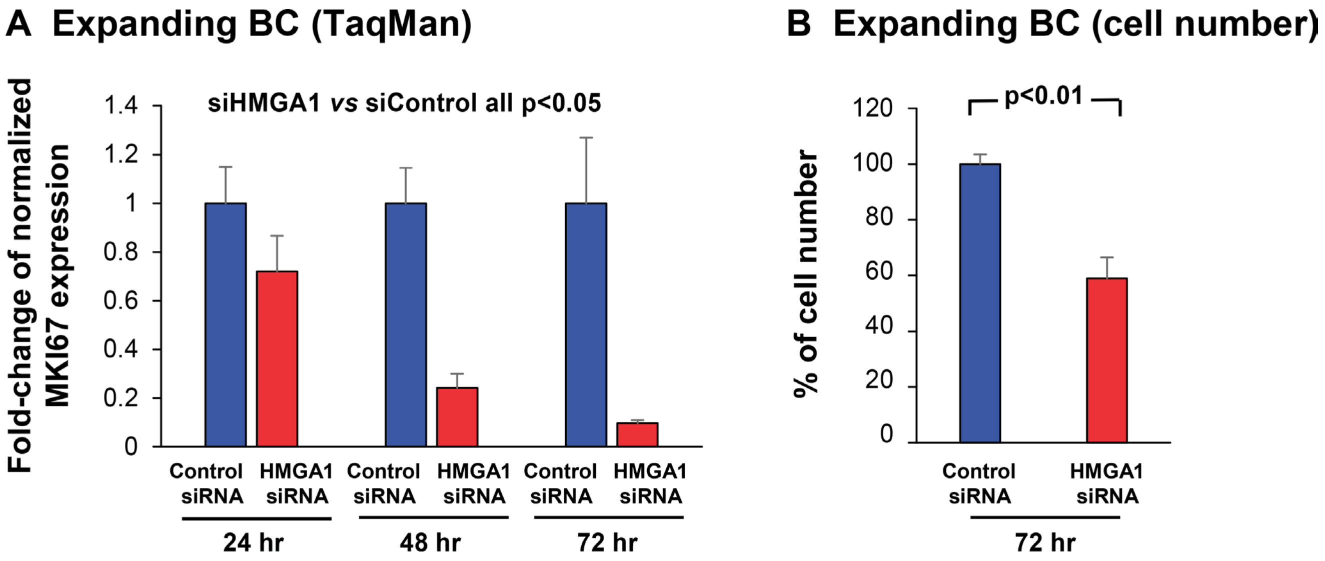

Supplementary Figure 3: Consequences of HMGA1 silencing on BC proliferation in expanding airway BC. BC were transfected with HMGA1 or control siRNA. Assessment was carried as time indicated. (**A**) Effect of HMGA1 silencing on marker of proliferation Ki-67 (MKI67) gene expression in expanding BC. Gene expression was assessed by TaqMan 24, 48, 72 hr after transfection. Data was normalized to 18S rRNA. Gene expression in control siRNA transfected samples was normalized to 1. *n* = 4, each group. LAE BC from Lonza, see Methods, BC-1. (**B**) Effect of HMGA1 silencing on BC cell number. Cell number was counted 72 hr after transfection. Cell number in control siRNA transfected samples was normalized to 100%. *n* = 3, each group. LAE BC from Lonza.

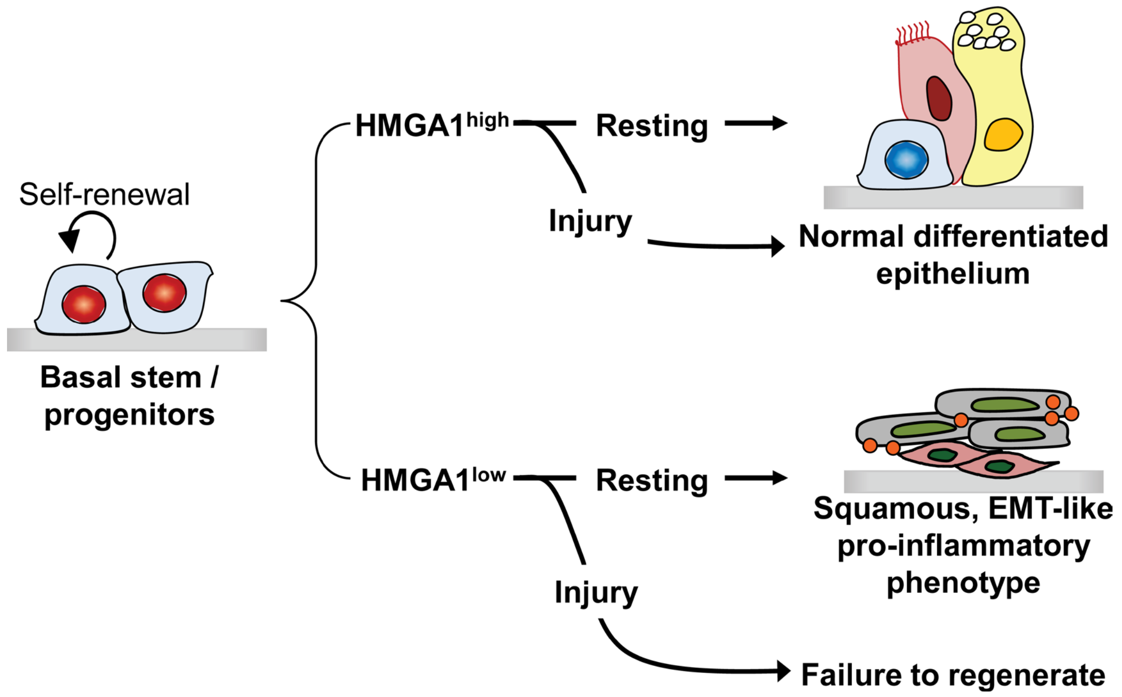

Supplementary Figure 4: A schematic summarizing HMGA1 function in airway epithelial. HMGA1 expression is critical to permit normal differentiation. If HMGA1 expression is low, BC differentiation defaults to a squamous, EMT-like, pro-inflammatory phenotype. Courtesy of R. Shaykhiev, Department of Medicine, Weill Cornell Medicine.
